# Supplementary figures and images for: The effect of de-escalation of P2Y12 receptor inhibitor therapy after acute myocardial infarction in patients undergoing percutaneous coronary intervention: A nationwide cohort study
Source: PLoS One. 2021 Jan 25;16(1):e0246029. doi: 10.1371/journal.pone.0246029 (PMC7833092; doi:10.1371/journal.pone.0246029)

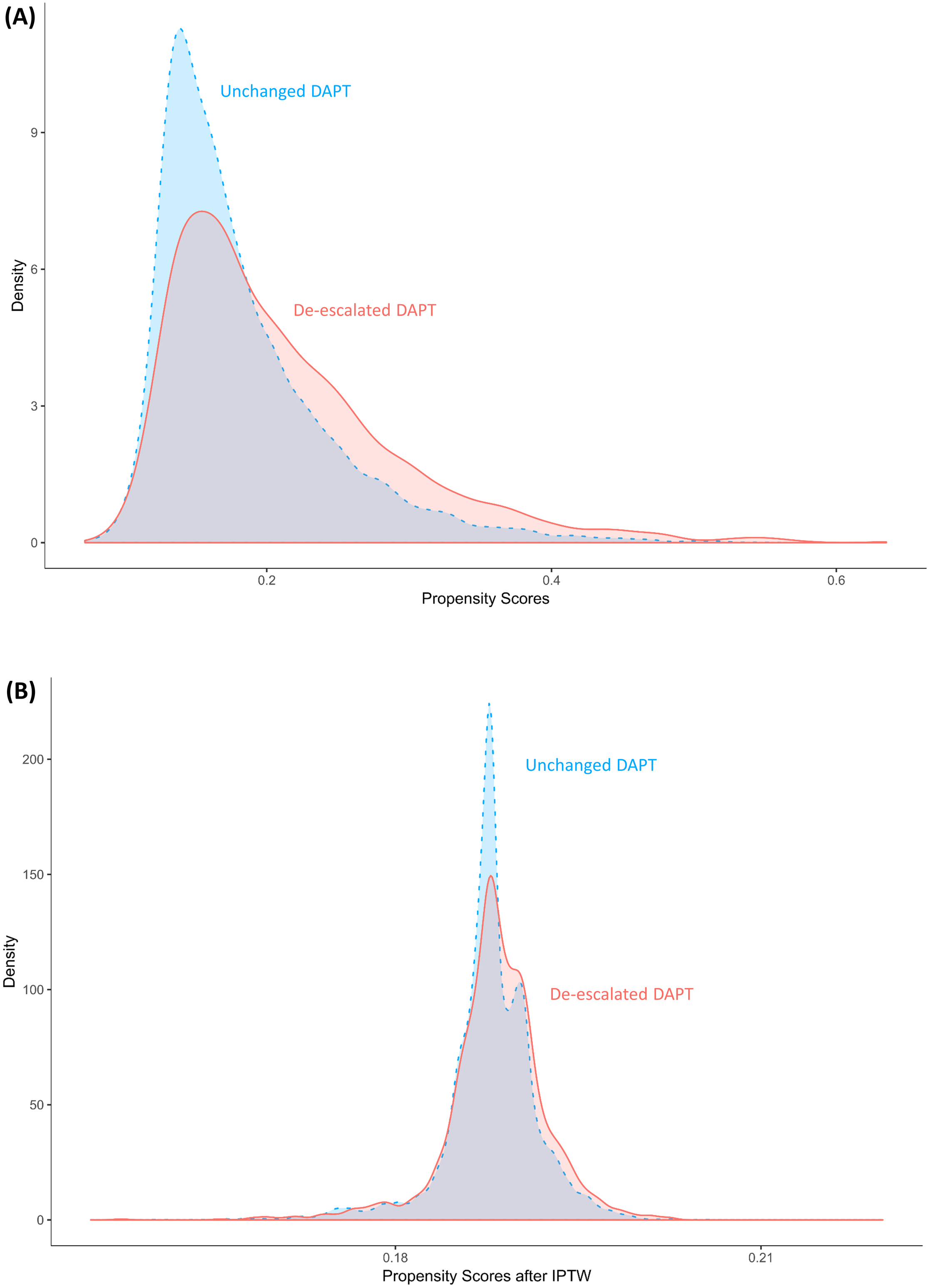

Supplement: S1 Fig — (TIF) [file pone.0246029.s001.tif]
