# Supplementary material for: The effect of de-escalation of P2Y12 receptor inhibitor therapy after acute myocardial infarction in patients undergoing percutaneous coronary intervention: A nationwide cohort study
Source: PLoS One. 2021 Jan 25;16(1):e0246029. doi: 10.1371/journal.pone.0246029 (PMC7833092; doi:10.1371/journal.pone.0246029)
Supplement: S1 Table — (DOCX) [file pone.0246029.s002.docx]

**Supplementary Table 1**. Disease diagnosis codes according to ICD-9-CM and ATC classification of medications

| Clinical outcomes or comorbidities | ICD-9-CM | ICD-10 |
| --- | --- | --- |
| Gastrointestinal bleeding | 456.0, 456.20, 530.7, 530.82, 531.0, 531.2, 531.4, 531.6, 532.0, 532.2, 532.4, 532.6, 533.0, 533.2, 533.4, 533.6, 534.0, 534.2, 534.4, 534.6, 535.01, 535.11, 535.21, 535.31, 535.41, 535.51, 535.61, 537.83, 562.02, 562.03, 562.12, 562.13, 569.3, 569.85, 578.0, 578.1, 578.9 | I8501, I8511, K226, K228, K250, K252, K254, K256, K260, K262, K264, K266, K270, K272, K274, K276, K280, K282, K284, K286, K2901, K2921, K2931, K2941, K2951, K2961, K2971, K2981, K2991, K31811, K3182, K5281, K5521, K5660, K5701, K5711, K5713, K5721, K5731, K5733, K5781, K5791, K5793, K625, K920, K921, K922 |
| Other non-critical site bleeding | 287.8, 287.9, 599.7, 596.7, 770.3, 784.7, 784.8, 786.3 | D698, D699, N3289, P260, P261, P268, P269, R040, R041, R042, R0481, R0489, R049, R310, R311, R312, R319 |
| Intracranial bleeding | 430, 431, 432, 432.0, 432.1, 432.9, 767.0, 852, 853 | I6000, I6001, I6002, I6010, I6011, I6012, I6020, I6021, I6022, I6030, I6031, I6032, I604, I6050, I6051, I6052, I606, I607, I608, I609, I610, I611, I612, I613, I614, I615, I616, I618, I619, I6200, I6201, I6202, I6203, I621, I629, P100, P101, P104, P108, P109, P110, P112, P119, P524, P526, P528, P529, S0190XA, S06340A, S06341A, S06342A, S06343A, S06344A, S06345A, S06346A, S06347A, S06348A, S06349A, S06350A, S06351A, S06352A, S06353A, S06354A, S06355A, S06356A, S06357A, S06358A, S06359A, S06360A, S06361A, S06362A, S06363A, S06364A, S06365A, S06366A, S06367A, S06368A, S06369A, S064X0A, S064X1A, S064X2A, S064X3A, S064X4A, S064X5A, S064X6A, S064X7A, S064X8A, S064X9A, S065X0A, S065X1A, S065X2A, S065X3A, S065X4A, S065X5A, S065X6A, S065X7A, S065X8A, S065X9A, S066X0A, S066X1A, S066X2A, S066X3A, S066X4A, S066X5A, S066X6A, S066X7A, S066X8A, S066X9A |
| Other critical site bleeding | 362.81, 363.61, 363.62, 376.32, 379.23, 423.0, 459.0, 568.81, 719.1 | H05231, H05232, H05233, H05239, H31301, H31302, H31303, H31309, H31311, H31312, H31313, H31319, H3560, H3561, H3562, H3563, H4310, H4311, H4312, H4313, I312, K661, M2500, M25011, M25012, M25019, M25021, M25022, M25029, M25031, M25032, M25039, M25041, M25042, M25049, M25051, M25052, M25059, M25061, M25062, M25069, M25071, M25072, M25073, M25074, M25075, M25076, M2508, R58 |
| Myocardial infarction | 410 | I2101, I2102, I2109, I2111, I2119, I2121, I2129, I213, I214, I220, I221, I222, I228, I229 |
| Ischemic stroke | 433, 434, 436 | I6300, I63011, I63012, I63019, I6302, I63031, I63032, I63039, I6309, I6310, I63111, I63112, I63119, I6312, I63131, I63132, I63139, I6319, I6320, I63211, I63212, I63219, I6322, I63231, I63232, I63239, I6329, I6330, I63311, I63312, I63319, I63321, I63322, I63329, I63331, I63332, I63339, I63341, I63342, I63349, I6339, I6340, I63411, I63412, I63419, I63421, I63422, I63429, I63431, I63432, I63439, I63441, I63442, I63449, I6349, I6350, I63511, I63512, I63519, I63521, I63522, I63529, I63531, I63532, I63539, I63541, I63542, I63549, I6359, I636, I638, I639, I6501, I6502, I6503, I6509, I651, I6521, I6522, I6523, I6529, I658, I659, I6601, I6602, I6603, I6609, I6611, I6612, I6613, I6619, I6621, I6622, I6623, I6629, I663, I668, I669, I6789 |
| Congestive heart failure | 428, 402.11, 402.91, 404.11, 404.13, 404.91, 404.93 | I110, I130, I132, I501, I5020, I5021, I5022, I5023, I5030, I5031, I5032, I5033, I5040, I5041, I5042, I5043, I509 |
| Peptic ulcer disease | 531 – 534 | K250, K251, K252, K253, K254, K255, K256, K257, K259, K260, K261, K262, K263, K264, K265, K266, K267, K269, K270, K271, K272, K273, K274, K275, K276, K277, K279, K280, K281, K282, K283, K284, K285, K286, K287, K289, K3182, K5660 |
| Hypertension | 401-405 | I10, I110, I119, I120, I129, I130, I1310, I1311, I132, I150, I151, I152, I158, I159, N262 |
| Diabetes | 250 | E0800, E0801, E08311, E08319, E08321, E08329, E08331, E08339, E08341, E08349, E08351, E08359, E0836, E0839, E0840, E0841, E0842, E0843, E0844, E0849, E0851, E0852, E0859, E08641, E088, E0900, E0901, E09311, E09319, E09321, E09329, E09331, E09339, E09341, E09349, E09351, E09359, E0936, E0939, E0940, E0941, E0942, E0943, E0944, E0949, E0951, E0952, E0959, E09641, E098, E1010, E1011, E1021, E1022, E1029, E10311, E10319, E1036, E1039, E1040, E1041, E1044, E1049, E1051, E1052, E1059, E10610, E10618, E10620, E10621, E10622, E10628, E10630, E10638, E10641, E10649, E1065, E1069, E108, E109, E1100, E1101, E1121, E1122, E1129, E11311, E11319, E11321, E11329, E11331, E11339, E11341, E11349, E11351, E11359, E1136, E1139, E1140, E1141, E1142, E1143, E1144, E1149, E1151, E1152, E1159, E11610, E11618, E11620, E11621, E11622, E11628, E11630, E11638, E11641, E11649, E1165, E1169, E118, E119, E1300, E1301, E1311, E1321, E1322, E1329, E13311, E13319, E13321, E13329, E13331, E13339, E13341, E13349, E13351, E13359, E1336, E1339, E1340, E1341, E1342, E1343, E1344, E1349, E1351, E1352, E1359, E13641, E138, E139 |
| Chronic liver disease | 571.2, 571.5, 571.6, 571.4, 572.2 – 572.8 | K702, K7030, K7031, K7210, K7211, K7290, K7291, K730, K731, K732, K738, K739, K740, K741, K742, K743, K744, K745, K7460, K7469, K754, K766, K767, K7681 |
| Hyperlipidemia | 272 | E780, E781, E782, E783, E784, E785 |
| COPD | 490 – 492, 494, 496 | J40, J410, J411, J418, J42, J430, J431, J432, J438, J439, J440, J441, J449, J470, J471, J479 |
| Valvular heart disease | 394 – 397, 424.0 – 424.2, 746.3 – 746.6 | I050, I051, I052, I058, I059, I060, I061, I062, I068, I069, I070, I071, I072, I078, I079, I080, I081, I082, I083, I088, I089, I091, I0989, I340, I341, I342, I348, I349, I350, I351, I352, I358, I359, I360, I361, I362, I368, I369, Q230, Q231, Q232, Q233 |
| Chronic kidney disease | 250.4, 274.1, 403, 404, 581 –583, 585–588 | E1021, E1022, E1029, E1065, E1121, E1122, E1129, E1165, E1321, E1322, E1329, I120, I129, I130, I1310, I1311, I132, M1030, M10311, M10312, M10319, M10321, M10322, M10329, M10331, M10332, M10339, M10341, M10342, M10349, M10351, M10352, M10359, M10361, M10362, M10369, M10371, M10372, M10379, M1038, M1039, N020, N021, N022, N023, N024, N025, N026, N027, N028, N029, N030, N031, N032, N033, N034, N035, N036, N037, N038, N039, N040, N041, N042, N043, N044, N045, N046, N047, N048, N049, N050, N051, N052, N053, N054, N055, N056, N057, N058, N059, N060, N061, N062, N063, N064, N065, N066, N067, N068, N069, N070, N071, N072, N073, N074, N075, N076, N077, N078, N079, N08, N140, N141, N142, N143, N144, N150, N158, N159, N16, N171, N172, N184, N185, N186, N189, N19, N200, N250, N251, N2581, N2589, N259, N261, N269 |
| Malignancy | 140—165, 170—176, 179–208, 230–234 | E1021, E1022, E1029, E1065, E1121, E1122, E1129, E1165, E1321, E1322, E1329, I120, I129, I130, I1310, I1311, I132, M1030, M10311, M10312, M10319, M10321, M10322, M10329, M10331, M10332, M10339, M10341, M10342, M10349, M10351, M10352, M10359, M10361, M10362, M10369, M10371, M10372, M10379, M1038, M1039, N020, N021, N022, N023, N024, N025, N026, N027, N028, N029, N030, N031, N032, N033, N034, N035, N036, N037, N038, N039, N040, N041, N042, N043, N044, N045, N046, N047, N048, N049, N050, N051, N052, N053, N054, N055, N056, N057, N058, N059, N060, N061, N062, N063, N064, N065, N066, N067, N068, N069, N070, N071, N072, N073, N074, N075, N076, N077, N078, N079, N08, N140, N141, N142, N143, N144, N150, N158, N159, N16, N171, N172, N184, N185, N186, N189, N19, N200, N250, N251, N2581, N2589, N259, N261, N26 |
| Intracerebral hemorrhage | 430-432 | I6000, I6001, I6002, I6010, I6011, I6012, I6020, I6021, I6022, I6030, I6031, I6032, I604, I6050, I6051, I6052, I606, I607, I608, I609, I610, I611, I612, I613, I614, I615, I616, I618, I619, I6200, I6201, I6202, I6203, I621, I629 |
| Medications | **ATC code** |  |
| Proton pump inhibitors | A02BC |  |
| HMG CoA reductase inhibitors | C10AA |  |
| Angiotensin-converting-enzyme inhibitors | C09AA, C09BA, C09BB, C09BX |  |
| Angiotensin II antagonists | C09CA, C09DA, C09DB, C09DX |  |
| β-blocker | C07 |  |
| Antiplatelet agents | B01AC |  |
| Anticoagulants | B01AA03, B01AE07, B01AF02, B01AF03, B01AF01 |  |
| Aspirin | B01AC06 |  |
| Ticagrelor | B01AC24 |  |
| Clopidogrel | B01AC04 |  |
