# Supplementary material for: The effect of de-escalation of P2Y12 receptor inhibitor therapy after acute myocardial infarction in patients undergoing percutaneous coronary intervention: A nationwide cohort study
Source: PLoS One. 2021 Jan 25;16(1):e0246029. doi: 10.1371/journal.pone.0246029 (PMC7833092; doi:10.1371/journal.pone.0246029)
Supplement: S2 Table — (DOCX) [file pone.0246029.s003.docx]

**Supplementary Table 2.**

| Supplementary Table 2. The incidence (per 100 PY) and adjusted HR of major vascular and bleeding events in unchanged DAPT versus de-escalation DAPT group during one-year follow-up. | | | | | | | |
| --- | --- | --- | --- | --- | --- | --- | --- |
| **Outcomes** | **DAPT Group** | **No. of Event** | **Incidence** | **(95% CI)** | **Adjusted* HR** | **(95% CI)** | **P-value** |
| AMI hospitalization | Unchanged | 105 | 2.18 | (1.79-2.62) | 1.00 | (Ref.) |  |
|  | De-escalation | 33 | 2.65 | (1.87-3.67) | 1.20 | (0.80-1.79) | 0.373 |
| MACE | Unchanged | 170 | 3.54 | (3.03-4.09) | 1.00 | (Ref.) |  |
|  | De-escalation | 44 | 3.47 | (2.54-4.60) | 0.98 | (0.69-1.37) | 0.885 |
| Major bleeding | Unchanged | 78 | 1.62 | (1.28-1.99) | 1.00 | (Ref.) |  |
|  | De-escalation | 19 | 1.51 | (0.96-2.34) | 0.95 | (0.58-1.56) | 0.849 |
| Non-major clinically relevant bleeding | Unchanged | 398 | 8.43 | (7.63-9.28) | 1.00 | (Ref.) |  |
|  | De-escalation | 105 | 8.54 | (7.00-10.3) | 1.04 | (0.84-1.29) | 0.721 |

* Adjusted HR was estimated by Cox proportional regression controlling for covariates listed in Table 1.

Abbreviation: CI=confidence interval, HR=hazard ratio, MACE=major adverse cardiovascular event
