# Supplementary material for: The effect of de-escalation of P2Y12 receptor inhibitor therapy after acute myocardial infarction in patients undergoing percutaneous coronary intervention: A nationwide cohort study
Source: PLoS One. 2021 Jan 25;16(1):e0246029. doi: 10.1371/journal.pone.0246029 (PMC7833092; doi:10.1371/journal.pone.0246029)
Supplement: Attachment — Submitted filename: Response to reviewers_r3.docx [file pone.0246029.s004.docx]

**Response to reviewers**

Reviewer #1: Yeh et al. report the impact of the de-escalation of P2Y12 inhibitors (i.e. ticagrelor to clopidogrel) on clinical outcomes among patients with STEMI. Among 10,100 STEMI patients (1901 with de-escalation and 8199 without de-escalation) from the Taiwan’s National Health Insurance Research Database, after IPTW adjustment, there were no significant differences in death (HR 1.20, 95% CI 0.83-1.73), AMI readmission (HR 1.12, 95% CI 0.80-1.56), MACE (HR 1.04, 95% CI 0.78–1.39), and major bleeding (HR 0.92, 95% CI 0.67-1.37) between groups. This reviewer has the following comments:

1. The clinical circumstance and reason for switching, relevant factors for patients’ outcome, were not reported in the current study. Considering that some of the clinical events (e.g. bleeding) triggered switching, only events occurred after switching should be considered for analysis (at least as a sensitivity analysis) to assess the causal relationship between the de-escalation and subsequent clinical events.

**Response:** Thank you for your comment and suggestion. This current study was aimed to study the subsequent outcomes after de-escalating of P2Y12 inhibitors among STEMI patients. We understood that several clinical circumstance and reasons for switching were unable to capture in the claim-based study although we used the propensity score technique to reduce the potential bias. We addressed this limitation in the revised manuscript. We also provided a sensitivity analysis that included the patients occurred an event of bleeding before switching. We can only identify 117 patients (<10% of switch group) had a diagnostic code of bleeding before de-escalation and their bleeding risk was significantly higher than those without de-escalation of P2Y12 inhibitors while no different in the risk of AMI admission and MACE.

Page 29, line 264 Considering that some of the clinical events (e.g. bleeding) triggered switching, we conducted a sensitivity analysis that only included the patients occurred an event of bleeding before switching. We can only identify 117 patients (<10% of de-escalation group) had a diagnostic code of bleeding before de-escalation and their bleeding risk was significantly higher than those without de-escalation of P2Y12 inhibitors while no different in the risk of AMI admission and MACE (See appendix table 3).

2. No significant decrease in bleeding events in the de-escalation group may be at least partly due to the patient characteristics not captured in the administrative database (e.g. frailty, risk profile change after index AMI). Several important unmeasured confounding factors due to the inherent limitation of administrative database may hinder to draw a definitive conclusion.

**Response:** Thanks for the comments. We recognized that some factors associated with de-escalation of P2Y12 inhibitors and outcomes were unmeasured due to the inherent limitation of administrative database. These factors might be biased our finding. We mentioned the limitation in the discussion section.

Page 33, line 319. Limitations. Although this study used propensity score technique to balance the baseline difference between two groups, several important unmeasured confounding factors, as such frailty of patients, were unable to control due to the inherent limitation of administrative database. Thus, a hidden bias of medication selection was introduced.

Methods

3. Why did the authors exclude patients treated with DAPT with prasugrel?

**Response:** We did not include the patients treated with DAPT with prasugrel because this medication was not approval for reimbursement under the regulation of National Health Insurance in Taiwan during the study period (here is from Jan 2013 to Dec 2016). Thus, we did not consider to include the patients treated with DAPT with prasugrel.

Page 6, line 105. The patients treated with prasugrel were not included because it had not been approved for reimbursement under the regulation of National Health Insurance during the study period.

4. Is there validation data on AMI readmission, CV death, and bleeding in the Taiwan’s National Health Insurance Research Database? If not available, please comment on the accuracy of data.

**Response:** Thanks for your suggestion. The accuracy of diagnostic code of AMI admission has been validated with a high PPV of 0.88 in any diagnosis based on NHIRD (Cheng et al., 2014). However, we did not find the validation data of bleeding. Fortunately, a validation study conducted in Korea found that the PPV of primary diagnostic code of GI bleeding in Health Insurance Claim was over 90%, showing favorable reliability (Park et al., 2019). The data of CV death was derived from National Death Registry (NDR). The completeness and accuracy of death records in Taiwan were high(Lu, Lee, & Chou, 2000), as it is mandatory to register all causes of death in the NDR. We added the information regarding the accuracy of data in the method section.

Page 8, line 148. The accuracy of diagnostic code of AMI admission has been validated with a high PPV of 0.88 in any diagnosis based on NHIRD (Cheng et al., 2014). In this current study, we additionally combined antiplatelet therapy to confirm the AMI diagnosis was active. The accuracy of bleeding diagnosis has not been validated in NHIRD; however, a validation study conducted in Korea found that the PPV of primary diagnostic code of GI bleeding in Health Insurance Claim was over 90%, showing favorable reliability(Park et al., 2019). The data of CV death was derived from National Death Registry (NDR). The completeness and accuracy of death records in Taiwan were high (Lu et al., 2000), as it is mandatory to register all causes of death in the NDR.

5. Please explain why the 3-month window was chosen.

**Response:** Thanks for your suggestion. The timing of de-escalating of P2Y12 inhibitors mainly depends on the treatment scheme after AMI discharge. A 3-month window was chosen because we can observe at least two outpatient visits in the period, allowing de-escalating of P2Y12 inhibitors if necessary.

Page 6, line 108. In the analytic cohort, a 3-month window was used to identify whether their DAPT treatment scheme was switched from ticagrelor plus aspirin to clopidogrel plus aspirin. A 3-month window was chosen because we can observe at least two outpatient visits in the period, allowing de-escalating of P2Y12 inhibitors if necessary.

6. Please provide the rational for the exclusion of CABG patients.

**Response:** The exclusion of CABG patients was because these patients usually were not treated DAPT with either ticagrelor or clopidogrel in our study period (2013-2016). As we know, the ACC/AHA guideline (Levine et al., 2016) focused update provided a class I recommendation for the resumption of DAPT after CABG in patients with acute coronary syndrome based on level C evidence in 2016. Despite this, the DAPT prescription in discharged patients with ACS post-CABG is still low and varied. Thus, we excluded those patients to increase the homogeneity in study sample. The rational for the exclusion of CABG was provided in the revised manuscript.

Page 6, line 99. 3) patients had undergone a coronary artery bypass graft during the study period because the DAPT prescription with either ticagrelor or clopidogrel in these patients were low despite the guidelines recommended (Levine et al., 2016). We therefore excluded these patients to increase the homogeneity of the study sample.

7. IPTW adjustment: Was propensity score used for the IPTW adjustment? Medication possession ratio may include data after switching from ticagrelor to clopidogrel, which should not be used for the calculation of propensity score. Please provide the distribution of propensity score between 2 groups.

**Response:** Thanks for your comment and suggestion. The distribution of propensity score between 2 groups is provided in the revised manuscript in the supplementary material. The propensity score was used for the IPTW adjustment in this current study. We considered the medication possession ratio (MPR) within three months into the propensity score because the efficacy of medication can be associated the subsequent health outcomes.

8. Definition of AMI readmission “the patient receiving a primary or secondary diagnosis of AMI after the index of AMI admission”: Including secondary diagnosis of AMI may limit the accuracy of AMI readmission compared with including only a primary diagnosis of AMI readmission.

**Response:** Thanks for your suggestion. In the current study, we used any diagnosis of AMI admission because the accuracy of diagnosis was high (Cheng et al., 2014). As suggested, we conducted a sensitivity analysis that defined the occurrence of an event of interest based on the primary diagnosis only. The results were consistent with our main finding and we added the information in the revised manuscript.

Page 26, line 221. To increase the validity of the study, we also performed sensitivity analyses that defined the occurrence of an event of interest based on primary diagnosis only. The results were consistent with the main findings (See supplementary Table 2).

9. Please provide the definition of CV death.

**Response:** The definition of CV death has been included in the Supplementary Table 1.

10. Please explain the rationale for using SMD to assess differences between groups instead of P values.

**Response:** Thanks for your suggestion. Standardized mean difference (SMD) is the most commonly used statistic to examine the balance of covariate distribution between treatment groups in the propensity score analysis. Because SMD is independent of the unit of measurement, it allows comparison between variables with different unit of measurement(Austin, 2010). Thus, we provided the rationale in the revised manuscript.

Page 18, line 158. Baseline characteristics were analyzed using standardized mean differences (SMD). SMD is the most commonly used statistic to examine the balance of covariate distribution between treatment groups in the propensity score analysis. Because SMD is independent of the unit of measurement, it allows comparison between variables with different unit of measurement. An SMD of >0.1 indicated the presence of a non-negligible difference between the two groups(Austin, 2010).

Results

11. It seems to be not necessary to show baseline characteristics after IPTW adjustment.

**Response:** Thanks for your comment. Since we would like to show the baseline characteristic between two groups can be balanced after the IPTW. Thus, we would like to present the baseline difference before and after IPTW.

12. Please provide the mean follow-up duration.

**Response:** The data of the mean follow-up duration has been reported in Table 1 in the revised manuscript.

13. Please provide the clinical outcomes as a table.

**Response:** The data of the clinical outcomes has been reported in Table 2 in the revised manuscript (see Page 24-Page 25).

Reviewer #2: Jong Shiuan Yeh et al. addressed the highly interesting field of de-escalating potent P2Y12 inhibitors in acute myocardial infarction patients post-PCI. The presented data is of high quality and contains more than 10’000 patients of which 1’901 switched to clopidogrel and 8’199 remained on ticagrelor. The Authors found no difference in ischemic events (all cause death, AMI readmission, and MACE) within one year follow up. Similarly, the incidences of major bleeding between the two groups was not different. The authors concluded correctly that a more detailed investigation is needed to answer the present question, yet the contemporary trial suggest no harm nor benefit. The Authors should be congratulated for their nice and accurate work. The Present Reviewer has following questions.

Major

1. The time point of de-escalation is crucial since the ischemic risk is highest during the first 30 days. How was this implemented in the present analysis

**Response:** Thank you for your comment. We understand that the time point of de-escalation is important in this study. Unlike the RCT, it is difficult to define the exact time of DAPT de-escalation in the claim-based research. In our study, a 3-month window, which covered at least 2 outpatient visits, was used to identify whether their DAPT treatment scheme was switched from ticagrelor plus aspirin to clopidogrel plus aspirin. We have made some description in the discussion section.

Page 30, line 282. The time point of de-escalation is crucial since the ischemic risk is highest during the first 30 days of index event. A retrospective study that included 1,019 ACS patients with de-escalation P2Y_12_ inhibitor treatment strategy showed that early de-escalation from ticagrelor to clopidogrel during the initial 30 days after ACS had an increased risk of ischemic events compared with switching beyond 30 days. [21]In this claim-based study, a 3-month window, which covered at least 2 outpatient visits, was used to identify whether their DAPT treatment scheme was switched from ticagrelor plus aspirin to clopidogrel plus aspirin. Based on our data (see KM curve figure 2A), we did not observe the risk of AMI admission within 30 days in the de-escalation group. Since the time point of de-escalation is very important for patients de-escalating potent P2Y12 inhibitors, future study is worth to investigate.

2. Different Reasons for de-escalation inflict different clinical scenarios and hitherto different risk. Was an investigation into reasons for de-escalation performed?

**Response:** Thanks for your comments. There are several clinical circumstance and reasons for de-escalation P2Y12 inhibitors that were unable to capture in the claim-based study. We acknowledged it in the discussion section.

Page 28 line 244. De-escalation from ticagrelor to clopidogrel occurs with a variable frequency in real-life practice that cannot be fully addressed in this current study According to some clinical registries data, approximately 50% of ticagrelor cessation were the result of ticagrelor-specific adverse effects, and the remainder included various clinical scenarios, such as initiation of oral anticoagulation, unspecified preference of the treating physician, or financial reasons. {Fosbøl, 2016 #113} {Zanchin, 2018 #104}

3. There is an ongoing discussion about ticagrelor and dyspnea and its impact on real world adherence. Would the authors be able to address this question?

**Response:** In this current study, we were unable to address the association between ticagrelor and dyspnea since the side effect of medication is usually underestimated in claim database. Besides, it is very difficult to distinguish the risk of dyspnea was due to medication use or disease condition itself. We would like to address the issue in the discussion section in the revised manuscript.

Page 32, line 301. In the ticagrelor-specific adverse effect, dyspnea is a second well-known side effect after bleeding. Nevertheless, rates of cessation because of dyspnea amounted to 5% in stable patients treated in phase III off-label trails {Hiatt, 2017 #108} {Bonaca, 2015 #107} and around 1% among patients with ACS in the randomized controlled trial and clinical registry {Zanchin, 2018 #104} , possibly because minor symptoms are better tolerated or may be attributed to other causes like cardiac or pulmonary cause than drug related effects in the early phase of an ACS{Granger, 2016 #109}. Unfortunately, this current study was unable to address the association between ticagrelor and dyspnea.

4. Why were patients who died within the first 3 months after hospital discharge excluded?

**Response:** We excluded the patients who died within the first 3 months after hospital discharge since their disease conditions and comorbidities were more likely to be complicated, resulting in the treatment scheme unlikely following the recommended guidelines. Besides, we used a 3-month window to group patients into de-escalation cohort, resulting in being unable to classify these patients. Thus, we excluded this population.

Page 8 line 102. The later exclusion was made because their disease condition and comorbidities were more likely to be complicated; thus, their treatment were less likely to follow the recommended guidelines. Besides, we used a 3-month window to group patients into de-escalation cohort, resulting in being unable to classify these patients. Thus, we excluded this population.

5. The SCOPE registry should be discussed in view of the present findings since it found higher events after de-escalation.

**Response:** Thanks for your comment. We added the discussion in the revised manuscript.

Page 29, line 257. Meanwhile, the SCOPE registry (Switching From Clopidogrel to New Oral Antiplatelet Agents During Percutaneous Coronary Intervention), a prospective single center observational study with 1363 ACS patients, suggested that de-escalation of antiplatelets early within 1 month after the index event in patients with acute coronary syndrome was associated with an increased risk of ischemic events (OR = 5.3; 95% CI: 2.1-18.2; p = 0.04) with no differences in bleeding at 1-month follow up. However, this population-based real-world data found that de-escalation from ticagrelor to clopidogrel 3 months after the index event of acute coronary syndrome was not associate with poor cardiovascular outcomes. The differences can be explained due to the heterogeneity of data source, study population, study design and so on.

6. The SWAP-4 trial, which assesses drug interaction due to de-escalation from ticagrelor to clopidogrel should be discussed.

**Response:** Thank for your comment. The SWAP-4 trial has been summarized and discussed in the revised manuscript.

Page 29, line 254. A pharmacodynamic study (SWAP-4) showed that de-escalation from ticagrelor to clopidogrel therapy was associated with an increase in platelet activity suggestive of a drug-drug interaction in the patient with stable coronary artery disease.

Minor

7. Number of patients at risk in the figures should be included

**Response:** This study used IPTW approach to adjust the baseline difference between two groups. The propensity score is used as a weight index; as a result, the number of sample size in each group was not an integer and the sample size were not the same as in two study cohorts before matching. Thus, we decided not to report the numbers at risk to avoid the confusing.

Austin, P. C. (2010). The performance of different propensity-score methods for estimating differences in proportions (risk differences or absolute risk reductions) in observational studies. *Stat Med, 29*(20), 2137-2148. doi:10.1002/sim.3854

Cheng, C.-L., Lee, C.-H., Chen, P.-S., Li, Y.-H., Lin, S.-J., & Yang, Y.-H. K. (2014). Validation of acute myocardial infarction cases in the national health insurance research database in taiwan. *Journal of Epidemiology, 24*(6), 500-507. doi:10.2188/jea.je20140076

Levine, G. N., Bates, E. R., Bittl, J. A., Brindis, R. G., Fihn, S. D., Fleisher, L. A., . . . Wijeysundera, D. N. (2016). 2016 ACC/AHA guideline focused update on duration of dual antiplatelet therapy in patients with coronary artery disease: A report of the American College of Cardiology/American Heart Association Task Force on Clinical Practice Guidelines. *J Thorac Cardiovasc Surg, 152*(5), 1243-1275. doi:10.1016/j.jtcvs.2016.07.044

Lu, T. H., Lee, M. C., & Chou, M. C. (2000). Accuracy of cause-of-death coding in Taiwan: types of miscoding and effects on mortality statistics. *Int J Epidemiol, 29*(2), 336-343. doi:10.1093/ije/29.2.336

Park, J., Kwon, S., Choi, E.-K., Choi, Y.-j., Lee, E., Choe, W., . . . Oh, S. (2019). Validation of diagnostic codes of major clinical outcomes in a National Health Insurance database. *International Journal of Arrhythmia, 20*(1), 5. doi:10.1186/s42444-019-0005-0
