# Supplementary material for: The effect of de-escalation of P2Y12 receptor inhibitor therapy after acute myocardial infarction in patients undergoing percutaneous coronary intervention: A nationwide cohort study
Source: PLoS One. 2021 Jan 25;16(1):e0246029. doi: 10.1371/journal.pone.0246029 (PMC7833092; doi:10.1371/journal.pone.0246029)
Supplement: Attachment — Submitted filename: response to reviewer210105_LN.docx [file pone.0246029.s005.docx]

In the first revision round, this reviewer asked to provide the results of IPTW analysis for endpoints occurred after de-escalation of DAPT (i.e. patients with any events occurred before de-escalation should be censored) to assess the impact of the de-escalation on the subsequent clinical events. However, the authors provided the results in patients with bleeding before de-escalation. And it is obvious that those patients had a higher bleeding risk than those without de-escalation. If time points of events and DAPT de-escalation are available in the dataset, please provide aforementioned analysis instead of supplementary table 3.

Response: Thank for the comment. We are sorry for the misinterpretation of the reviewer’s suggestion in the last revision. In the study, the endpoints of any events were after de-escalation of DAPT. We have made more clarification in the method section and we had deleted the paragraph about supplementary table 3.

Page 8 line 147-149.

This study assessed the effect of DAPT de-escalation strategy on the subsequent endpoints, therefore the patients with any event occurred before the 3 months DAPT de-escalation time point was censored.

- For better understanding of the readers, the definition of grouping should be clearly mentioned in the Method section (e.g. patients were considered the de-escalation group if ticagrelor was changed to clopidogrel 3 months after index AMI hospitalization).

Response: Thank for the suggestion. We have made the definition clearer in the revision.

Page 6 line 112-113.

The patients were considered as the de-escalation group if ticagrelor was changed to clopidogrel within 3 months after index AMI hospitalization.
